# Physiological and Transcriptional Responses of *Streptomyces albulus* to Acid Stress in the Biosynthesis of ε-Poly-L-lysine

**DOI:** 10.3389/fmicb.2020.01379

**Published:** 2020-06-19

**Authors:** Chenying Wang, Xidong Ren, Chao Yu, Junming Wang, Li Wang, Xin Zhuge, Xinli Liu

**Affiliations:** ^1^State Key Laboratory of Biobased Material and Green Papermaking, Qilu University of Technology, Shandong Academy of Sciences, Jinan, China; ^2^Shandong Provincial Key Laboratory of Microbial Engineering, Department of Bioengineering, Qilu University of Technology, Shandong Academy of Sciences, Jinan, China; ^3^Process Development Department, IntellectiveBio Co., Ltd., Suzhou, China

**Keywords:** *Streptomyces albulus*, ε-poly-L-lysine, acid stress, acid tolerance response, RNA-sequencing

## Abstract

*Streptomyces albulus* has commercially been used for the production of ε-poly-L-lysine (ε-PL), a natural food preservative, where acid stress is inevitably encountered in the biosynthesis process. To elucidate the acid tolerance response (ATR), a comparative physiology and transcriptomic analysis of *S. albulus* M-Z18 at different environmental pH (5.0, 4.0, and 3.0) was carried out. In response to acid stress, cell envelope regulated the membrane fatty acid composition and chain length to reduce damage. Moreover, intracellular pH homeostasis was maintained by increasing H^+^-ATPase activity and intracellular ATP and amino acid (mainly arginine, glutamate, aspartate and lysine) concentrations. Transcriptional analysis based on RNA-sequencing indicated that acid stress aroused global changes and the differentially expressed genes involved in transcriptional regulation, stress-response protein, transporter, cell envelope, secondary metabolite biosynthesis, DNA and RNA metabolism and ribosome subunit. Consequently, the ATR of *S. albulus* was preliminarily proposed. Notably, it is indicated that the biosynthesis of ε-PL is also a response mechanism for *S. albulus* to combat acid stress. These results provide new insights into the ATR of *S. albulus* and will contribute to the production of ε-PL via adaptive evolution or metabolic engineering.

## Introduction

ε-Poly-L-lysine (ε-PL) is a homopolymer of 25-35 L-lysine residues with amide linkage between ε-amino and α-carboxyl groups (Shima and Sakai, 1977). It is biodegradable, water-soluble, heat-stable and exhibits widely antimicrobial spectra, including yeast, fungi, Gram-positive and Gram-negative bacteria, as well as antiphage activity. Moreover, ε-PL also shows excellent behavior in high safety. Therefore, ε-PL has been widely used as a natural food preservative in many countries, including Japan, Korea and the United States as well as China (Ren et al., 2015).

To date, commercial ε-PL production is mainly based on microbial fermentation by *Streptomyces albulus* which belongs to actinomycetes. As is known, actinomycetes is Gram-positive and the optimum pH for growth is neutral or alkalescent. However, the producing bacteria face acid stress in the biosynthesis of ε-PL: environmental pH spontaneously decreased from initial 6.8 to 3.0 during fermentation, while ε-PL production could only be detected when pH was below 5.0 and the maximum synthesis rate was obtained at about pH 4.0; moreover, the cells still retained a certain level of metabolic activity even at pH 3.0 (Kahar et al., 2001; Ren et al., 2015). Generally, it is believed that an acidic environment can lead to the decrease of intracellular pH (pH_i_), inactivation of acid-sensitive enzymes in the glycolytic pathway and structural damage of the cell membrane, intracellular macromolecules such as DNA and proteins, and thereby causing cell death (Cotter and Hill, 2003; Lund et al., 2014). Therefore, the ε-PL-producing strains exhibit acid tolerance, and it is very important to study the acid tolerance response (ATR) of the *S. albulus*.

In response to acid stress, the Gram-positive bacteria, e.g., *Lactobacillus* and *Bacillus*, employ a combination of constitutive and inducible strategies to counter the acidic environment, including alkalization of external environment, alterations in cell envelope, maintenance of pH_i_, expression of transcriptional regulators and production of general shock proteins and chaperones (Broadbent et al., 2010; Senouci-Rezkallah et al., 2011; Wu et al., 2012a; Lund et al., 2014; Ter Beek et al., 2015). Nevertheless, despite of the previous work on Gram-positive bacteria, the ATR of *S. albulus* has not been studied so far. In the present work, a comparative study on the physiological and transcriptional responses of *S. albulus* M-Z18, a ε-PL-producing strain, at different environmental pH for ε-PL biosynthesis (the highest pH 5.0, the optimum pH 4.0 and the lowest pH 3.0) was conducted to elucidate the ATR of *S. albulus* in the biosynthesis of ε-PL. To our knowledge, this is the first attempt to disclose the ATR of *S. albulus*.

## Materials and Methods

### Microorganism and Inoculum Preparation

*Streptomyces albulus* M-Z18 was used throughout this study, which was a mutagenesis from *S. albulus* Z-18 (CGMCC 10479). Agar slant medium, used to maintain the strain, composed of (g/L): glucose, 10; yeast extract, 5; beef extract, 5; MgSO_4_⋅7H_2_O, 0.5; K_2_HPO_4_⋅3H_2_O, 1; and agar 20, along with pH 7.0 before sterilization. Seed culture medium (M3G), contained (g/L): glucose, 50; yeast extract, 5; (NH_4_)_2_SO_4_, 10; KH_2_PO_4_, 1.36; K_2_HPO_4_⋅3H_2_O, 0.8; MgSO_4_⋅7H_2_O, 0.5; ZnSO_4_⋅7H_2_O, 0.04; FeSO_4_⋅7H_2_O, 0.03. Fermentation medium containing (g/L): glycerol, 60; (NH_4_)_2_SO_4_, 5; beef extract, 10; KH_2_PO_4_, 4; MgSO_4_⋅7H_2_O 0.8; FeSO_4_⋅7H_2_O, 0.05. Initial pH values of the above two media were adjusted to 6.8 with 2 M NaOH and/or 1 M H_2_SO_4_. All the media were sterilized in an autoclave for 20 min at 121°C. In each case, glucose was autoclaved separately. The slants were inoculated and incubated at 30°C for 7 days to obtain a heavy sporulated growth. After that time, spores were used for seed-culture inoculation (in a concentration of about 2 × 10^5^ spores/mL). The seed culture was grown in a 500 mL Erlenmeyer flask containing 80 mL of liquid medium and incubated at 30°C on a rotary shaker (200 r/min) for 24 h.

### Batch Fermentation

**FIGURE 1:**
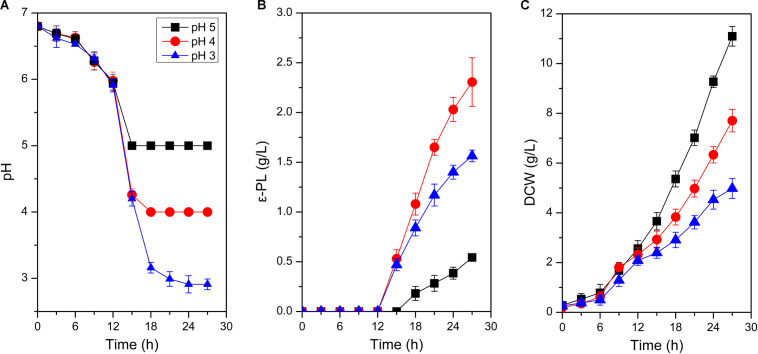
Time profiles of pH change **(A)**, ε-PL production **(B)**, and cell growth **(C)** in batch fermentations by *S. albulus* M-Z18 at different environmental pH (pH 5.0, 4.0, and 3.0). Error bars indicate the standard deviations from three parallel samples.

A 5-L fermenter (BIOTECH-5BG, BaoXing Bio-Engineering Equipment, China) with a 3.5-L working volume and two Rushton turbines (Φ = 6 cm) was employed for batch fermentation in this study. Before the inoculation, temperature, aeration rate and agitation speed were maintained at 30°C, 0.5 vvm and 200 rpm, respectively, and initial pH was controlled at 6.8 via manual addition of ammonia water (12.5%, w/v). Approximately 300 mL of seed culture was used as the inoculum. Dissolved oxygen (DO) was set above 30% of air saturation, which was controlled by manually adjusting agitation speed from 200 to 800 rpm and aeration rate with a range of 0.5-2.5 vvm. During the fermentation process, pH and DO were respectively monitored online by pH and DO electrodes (K8S-225 and InPro6800, Mettler Toledo, Switzerland). To investigate the ATR of *S*. *albulus* M-Z18, pH was respectively maintained at 5.0, 4.0 and 3.0 by ammonia water (12.5%, w/v) when it spontaneously dropped from initial 6.8 to the set values, and then the cells were harvested at 27 h (Figure 1A). At this time, it was about 12 hours since pH spontaneously dropped to 4.0, which was in accordance with the acidic-shock time in our previous study (Ren et al., 2015).

### Measurement of Dry Cell Weights and ε-PL Concentration

Ten milliliters of culture broth was subjected to centrifugation at 4,500 × *g* for 10 min, and then the precipitate was used to measure the dry cell weights (DCW) of the culture. The supernatant was used to determine the ε-PL concentration according to the procedure described by Itzhaki (1972).

### Scanning Electron Microscopy

Scanning electron microscopy (SEM) was used to observe the mycelia of *S*. *albulus* M-Z18 according to Shimada et al. (1993) with slight modification. Briefly, mycelia were first harvested by centrifugation at 4,500 × *g* for 10 min and washed twice with 0.1 M phosphate buffer (pH 7.0). After fixation with glutaraldehyde (2.5%, v/v) at 4^o^C for 4 h, the mycelia were washed thrice with the same phosphate buffer and then dehydrated with gradient ethanol solutions. Finally, the cells were freeze-dried and observed under a SEM (Quanta 200, FEI, United States).

### Observation of Cell Membrane Integrity

To observe the integrity of cell membrane, the LIVE/DEAD Bac-Light Bacterial Viability Kit L-13152 (Invitrogen detection technologies, United States) containing two nucleic acid staining dyes, propidium iodide (PI) and SYTO 9, was used. The SYTO9 is a green fluorescent stain which enters all the cells, those with intact membranes as well as those with damaged ones. In contrast, PI only penetrates dead cells with damaged membranes. However, PI has a higher affinity for nucleic acids and displaces SYTO 9 in dead cells. Therefore, in the presence of both stains, bacteria with intact cell membranes appear to fluorescent green, whereas bacteria with damaged membranes appear red (Rioseras et al., 2014). Biomass samples drawn from the bioreactor were centrifuged, washed twice and re-suspended with saline (0.9% NaCl) to about 10^5^-10^6^ pellets per mL. The two stains were prepared and mixed together (1:1, v/v) as recommended by the manufacturer. Equal volume (20 μL) of the stain mixture and culture samples was mixed on a clean slide and left in the dark for at least 10 min (Singh et al., 2013). Then, the sample covered with a cover slip, analyzed under a Leica confocal laser-scanning microscope (TCS-SP8, Leica Microsystems, Germany), was sequentially excited at wavelengths of 488 nm and 568 nm and observed at emission wavelengths of 530 nm (green) and 630 nm (red), respectively. A significant number of images were analyzed in a minimum of three independent culture analyses.

### Fatty Acids Extraction and Analysis

The extraction of fatty acids from cells and the subsequent determination were operated according to Sasser (1990). Mycelia collected by centrifugation (4,500 × *g* at 4^o^C for 10 min) were washed twice with saline (0.9% NaCl), and then sequentially processed by saponification, methylation, extraction and base wash. The top organic phase was used for GC-MS (TSQ Quantum XLS, Thermo Fisher Scientific, United States) determination.

### Measurement of Intracellular pH, H^+^-ATPase and ATP

Intracellular pH (pH_i_) was measured using 2’,7’-bis-(2-carboxyethyl)-5(and 6)-carboxyfluorescein acetoxymethyl ester (BCECF AM) as the fluorescent probe. The biomass sample grown at different pH values (3.0, 4.0 and 5.0) were harvest by centrifugation (4,500 × *g* at 4^o^C for 10 min) and washed thrice with 0.1 M phosphate buffer (pH 7.0). The wet mycelia were re-suspended with the same buffer and disrupted by ultrasonic (650 w, 2s/2s) in an ice bath. After removal of the unbroken mycelia by centrifugation (600 × *g* at 4^o^C for 4 min), the remaining hyphal fragments were used to determine the pH_i_. Incubation of cells with BCECF AM, calibration and determination of pH_i_ were done following the procedure described by Breeuwer et al. (1996).

The H^+^-ATPase activity was measured with the H^+^-ATPase assay kit (GENMED, China) following manufacturer’s protocol. The activity of the H^+^-ATPase was expressed in nanomoles of the NADH oxidized per minute per milligram protein.

Intracellular ATP was determined as described by Wu et al. (2012a). In brief, biomass sample extracted from the fermenter was immediately quenched by liquid nitrogen. Then, 0.6 M HClO_4_ was added in duplicate and both supernatants collected by centrifugation (12,000 × *g* at 4^o^C for 10 min) were blended. The mixture was adjusted to pH 7.0 with 1 M KOH and filtrated by a 0.22 μm membrane for HPLC determination.

### Intracellular Amino Acids Determination

Two milliliters of biomass sample were harvested by centrifugation at 4,500 × *g* for 10 min, washed thrice with ultrapure water. The cells were re-suspended with 1 mL of 10% trichloroacetic acid at 37^o^C for 10 min, and then boiled for 15 min. Cell debris were discarded by centrifugation (12,000 × *g* at 4^o^C for 10 min), and the supernatant was analyzed by HPLC according to the method of Fountoulakis and Lahm (1998).

### mRNA Sequencing and Transcriptome Analysis

For transcriptome analyses, 10 mL samples were separately withdrawn from three independent batch fermentations (biological replicates) at 27 h. These samples were immediately mixed together and quenched with liquid nitrogen for total RNA extraction. Total RNA was extracted using a RiboPure^TM^-Yeast Kit (Life technologies, United States). Further processing with DNase I (NEB, United States) was made to digest DNA and rRNA was also removed by Ribo-Zero^TM^ Magnetic Kit (Epicentre, United States) to reduce sequencing interference. The mRNA was interrupted to short fragments and reverse-transcribed into single-stranded cDNA. A double-stranded cDNA was synthesized in a double-stranded synthetic reaction system, which was sequentially purified with Agencourt RNAClean XP Kit (Beckman Coulter, United States), end repaired, d(A) added and ligated to Illumina sequencing adaptors. After that, suitable fragments were selected and PCR amplification was carry out. Finally, the constructed cDNA library was sequenced using Illumina HiSeq^TM^ 2000. The sequencing raw reads were filtered to discard adapters, unknown or low quality bases and clean reads were obtained.

These filtered clean reads were mapped to the complete genome of *S. albulus* ZPM (NCBI accession no. NZ_CP006871) with the employment of SOAPaligner/SOAP2. Differentially expressed genes (DEGs) with transcription differences more than 2-folds (p-values < 0.001, FDR < 0.001) under two comparison groups (pH 5.0 vs pH 4.0, pH 4.0 vs pH 3.0) were screened out, respectively.

### Quantitative Reverse Transcription-PCR (qRT-PCR) Validation

To ensure the reliability of RNA-sequencing data, 7 DEGs (*mprA*, *pepD*, *sigE*, *hrdD*, *pls*, *pld* and *htpX*) related to signal transduction, ε-PL synthesis and degradation, and stress response were verified by qRT-PCR. Total RNA was obtained as section 2.9. cDNA was synthesized using AMV First Strand cDNA Synthesis Kit (Sangon Biotech, China). The qRT-PCR was conducted in a ABI Stepone plus Real-time PCR instrument (Applied Biosystems, United States) and performed using a SG Fast qPCR Master Mix (High Rox) (Bio Basic, Canada) with a 20 μL system: 10 μL SybrGreen qPCR Master Mix (2X), 0.4 μL PCR forward primer (10 μM), 0.4 μL PCR reverse primer (10 μM), 7.2 μL ddH_2_O and 2 μL cDNA template. The parameters were: pre-incubation at 95^o^C for 3 min and 40 cycles of amplification step (melt at 95^o^C for 5 s, anneal 60^o^C for 10 s and extend at 72^o^C for 15 s). The 16S rDNA was used as endogenous reference gene. The qRT-PCR primers were designed using Primer Premier 5.0 (Supplementary Table S1). All experiments were repeated with at least three biological replicates.

### Statistical Analysis

To check the reproducibility, the experiments were carried out at least triplicate. The statistical significance of the data was determined by SPSS Statistics 20 (IBM, United States) using analysis of a one-way analysis of variance (ANOVA) followed by Tukey’s honestly significant difference (HSD) *post hoc* test (*p* ≤ 0.05).

### Data Availability Statement

RNA-seq data of *S. albulus* M-Z18 at different environmental pH values (pH 5.0, 4.0 and 3.0) were deposited at Sequence Read Archive (SRA) of National Center for Biotechnology Information (NCBI) under the accessions of SAMN14996498, SAMN14996497 and SAMN14996496, respectively.

## Results and Discussion

### Physiological Analysis

#### Growth Performance and ε-PL Production of *S. albulus* M-Z18 at Different Environmental pH

As shown in Figure 1, environmental pH could significantly affect cell growth and ε-PL production. Biomass gradually decreased when pH declined from 5.0 to 3.0. With the decline of environmental pH, DCW decreased from the maximum of 11.10 ± 0.39 g/L at pH 5.0 to the minimum of 4.98 ± 0.40 g/L at pH 3.0, with 55.14% decrease (Figure 1C). However, the influence of environmental pH on ε-PL production was much different from that of cell growth (Figure 1B). When pH was set at 4.0, ε-PL production reached the maximum of 2.31 ± 0.24 g/L, while the minimum ε-PL production of 0.54 ± 0.03 g/L was achieved at pH 5.0. These phenomena were in accordance with those observed in other ε-PL-producing strains (Kahar et al., 2001; Shih and Shen, 2006). To exemplify, Kahar et al. (2001) found that ε-PL production at pH 3.0, 4.0, 5.0, and 6.0 was 0.6, 8.2, 0.4, and 0.0 g/L, respectively. Likewise, cell growth was suppressed with the decrease of environmental pH. It could be concluded that environmental pH shows identical impact on cell growth and ε-PL production in the overall ε-PL-producing strains. Notably, ε-PL production by the unit biomass increased with the decline of environmental pH. The *Y*_ε –PL/DCW_ (the mass ratio of ε-PL to DCW) at pH 5.0, 4.0 and 3.0 was 0.05, 0.30, and 0.31 g/g DCW, respectively. Therefore, analyses on the response mechanisms of *S*. *albulus* M-Z18 to acid stress could not only enrich the content of ATR in Gram-positive bacteria but disclose why ε-PL production was promoted by acid stress.

#### Effects of Environmental pH on the Cell Envelope of *S. albulus* M-Z18

**FIGURE 2:**
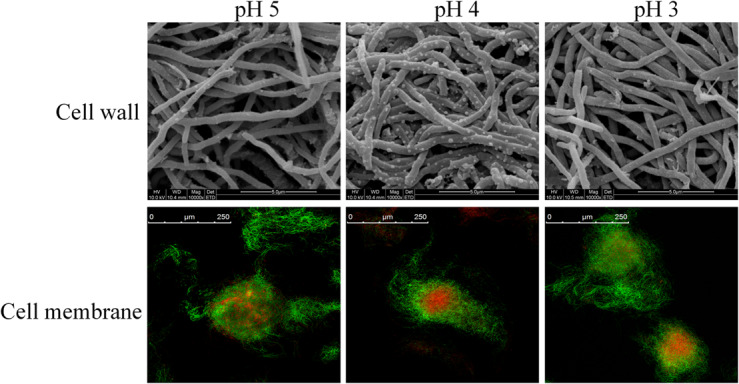
Scanning electron microscope and confocal laser scanning microscope stained by SYTO9 and PI of *S. albulus* M-Z18 in batch fermentations at different environmental pH (pH 5.0, 4.0, and 3.0). Samples were collected at 27 h.

Bacterial cell envelope, consist of cell wall and cell membranes, is an essential defensive barrier against various environmental stresses (Tran et al., 2019). Cell wall is the first barrier to contact with outside, which plays an important role against the adverse environment. Therefore, the effect of environmental pH on cell wall was first carried out. As shown in Figure 2, mycelia retained an intact and regular shape at different environmental pH (5.0, 4.0 and 3.0), indicating the integrity of cell wall structure and function. Consequently, it could provide the prerequisite for cell to maintain normal physiological metabolism under acid stress. Notably, when environmental pH was set at 4.0, vesicular protuberances emerged on mycelium surfaces. It is hypothesized that the vesicular protuberances might be related to the synthesis or secretion of secondary metabolites by *S. albulus*.

While cell wall shows little effect on permselectivity, the semipermeable cell membrane becomes the foremost barrier for cell to separate from outside. Cell membrane plays important roles in substance transport, energy metabolism, cellular growth and maintenance of a constant intracellular environment (Denich et al., 2003; Zhang and Rock, 2008). Structure integrity is the prerequisite for the function of cell membrane. Therefore, SYTO 9 and PI were first employed to observe the membrane integrity of *S*. *albulus* M-Z18 at different environmental pH. Figure 2 shows that the integrity of cell membrane scarcely changed with the decrease of environmental pH. This indicated that cell membrane maintained healthy structure and function even surrounded by acidic environment, which provided protection for the physiological function of cells. In addition, mycelia with red fluorescence, i.e., dead cells loss of membrane permeability, were found inside all of the pellets obtained at different environmental pH (5.0, 4.0, and 3.0). Cell death from the inside of pellets was a programmed process of mycelium differentiation in the submerged culture of *Streptomyces*, which was the prerequisite for the production of secondary metabolites (Manteca et al., 2008). Moreover, ε-PL production was found in the above three conditions, indicating that ε-PL production may also be caused by mycelium differentiation.

**FIGURE 3:**
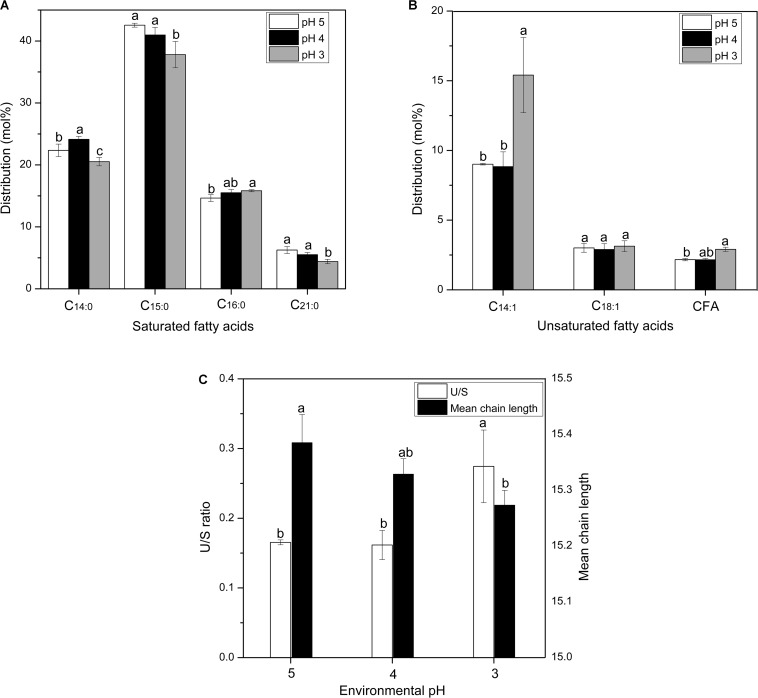
Changes in the distributions of membrane saturated fatty acid **(A)** and unsaturated fatty acid **(B)**, U/S ratio and mean chain length **(C)** of *S. albulus* M-Z18 in batch fermentations at different environmental pH (pH 5.0, 4.0, and 3.0). Samples were collected at 27 h. Statistical significance is denoted by different letter for the same indicator.

The regulation of membrane fatty acid composition is also an important approach for cells to combat acid stress (Denich et al., 2003). Moreover, the modulation of unsaturated and saturated fatty acid ratio (U/S ratio) and fatty acid chain-length could directly influence the liquidity and stability of the cell membrane (Russell, 1984). Figure 3 shows the alterations of saturated and unsaturated fatty acids distributions in the membrane of *S. albulus* M-Z18 under acid stress. It is observed that the membrane fatty acids of *S. albulus* M-Z18 mainly contained saturated fatty acids of myristic acid (C_14__:__0_), pentadecane acid (C_15__:__0_), palmitic acid (C_16__:__0_) and heneicosanoic acid (C_21__:__0_) and unsaturated fatty acids of myristoleic acid (C_14__:__1_), oleic acid (C_18__:__1_) and cyclopropane fatty acid (CFA). With the decrease of environmental pH, the contents of saturated fatty acids (C_14__:__0_, C_15__:__0_ and C_21__:__0_) decreased (Figure 3A), while the contents of unsaturated fatty acids (C_14__:__1_ and CFA) increased (Figure 3B). Notably, the most significant increase was found in the content of C_14__:__1_, which increased from the minimum of 9.01 ± 0.06% at pH 5.0 to the maximum of 15.41 ± 2.70% at pH 3.0, with 71.03% increase (Figure 3B). Besides, the increase of CFA could compact the cell membrane structure and prevent the invasion of harmful substances (Yin et al., 2019). Likewise, membrane CFA content was also found to be a major factor in the acid resistance of *Escherichia coli* (Chang and Cronan, 1999). As a result, the U/S ratio was increased when environmental pH declined from 5.0 to 3.0 (Figure 3C). The increased proportion of unsaturated fatty acids with a concomitant decrease in the proportion of saturated fatty acids in its membrane to combat acid stress was also reported in other bacteria (Fozo and Quivey, 2004; Wu et al., 2012b; Xu et al., 2020). Besides, the membrane fatty acid chain-length was reduced under acid stress (Figure 3C). In addition to fatty acid distribution, alteration of fatty acid chain-length is another important approach used by cells to increase survival in acidic environment (Guerzoni et al., 2001; Wu et al., 2012b). It was reported that shorter-chain fatty acids are hard to span the membrane bilayer and cannot form hydrophobic interactions with other lipids and proteins, resulting in increased fluidity of the cell membrane (Cao-Hoang et al., 2008). Consequently, substance transport and energy metabolism on the cell membrane was facilitated, which would guarantee the normal function of cells under acid stress. However, the decrease of fatty acid chain-length would reduce the stability of cell membrane, and thereby cell death was more easily happened in acidic environment.

#### Effects of Environmental pH on pH_i_, H^+^-ATPase Activity and Intracellular ATP Concentration

**FIGURE 4:**
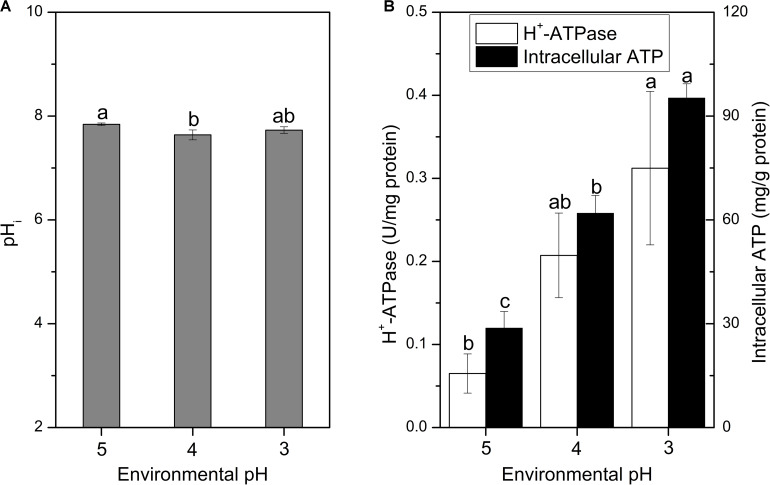
Changes in pH_i_ **(A)**, H^+^-ATPase and intracellular ATP concentration **(B)** of *S. albulus* M-Z18 in batch fermentations at different environmental pH (pH 5.0, 4.0, and 3.0). Samples were collected at 27 h. Statistical significance is denoted by different letter for the same indicator.

The pH_i_ plays an important role in the growth and metabolism of cells, and it can affect the uptake of nutrients, protein synthesis, glycolysis and synthesis of nucleic acids (Hutkins and Nannen, 1993; O’Sullivan and Condon, 1997). When suffered with acid stress, the pH_i_ of cells should maintain homeostasis, otherwise, protein and DNA damages would take place and finally lead to cell death (Budin-Verneuil et al., 2005). As the decline of environmental pH, the pH_i_ of cells slightly decreased, but still maintained at about 7.7 (Figure 4A). Cells of *S. albulus* M-Z18 seemed to have the ability to stabilize pH_i_, which is essential for the survival of cells during acid stress. Corvini et al. (2000) also used BCECF AM as a fluorescent probe to determine pH_i_, after image analysis by fluorescence microscopy, the pH_i_ of *S. pristinaespiralis* was disclosed ranging from 6.3 to 8.7. Although the methods used were different, but the resulting pH_i_ values were consistent with this study. In addition, Yamanaka et al. (2008) reported that ε-PL synthetase (Pls) is a membrane enzyme and the maximum activity of purified Pls occurred at an optimum pH of 8.5. *In vitro*, the enzyme activity was significantly inhibited with the decline of pH from 8.5, and the activity decreased to about 20% of relative activity at pH 6.8. However, the optimum environmental pH for ε-PL synthesis is about 4.0 (Kahar et al., 2001; Ren et al., 2015). Thus, there is a certain contradiction. In the present study, we found that even the environmental pH dropped to 4.0 or 3.0, the mycelia could still maintain pH_i_ at about 7.7. Therefore, the Pls could maintain about 65% of relative activity in the process of ε-PL biosynthesis according to the study by Yamanaka et al. (2008).

It is reported that pH_i_ homeostasis can be influenced by many factors, while the proton-translocating H^+^-ATPase plays the most important role; Meanwhile, the function of H^+^-ATPase requires ATP to provide energy to pump intracellular proton (Cotter and Hill, 2003; Lund et al., 2014). Figure 4B shows that the H^+^-ATPase activity and intracellular ATP concentration gradually increased with the decrease of environmental pH. Therefore, the mycelia maintained higher H^+^-ATPase activity and intracellular ATP concentration under lower environmental pH, so that the intracellular proton could be effectively pumped out of the cell to maintain the pH_i_ stable. Similarly, *Lactobacillus plantarum* could produce more ATP through glycolysis to enhance oxidative tolerance (Zhai et al., 2020). However, the increase of intracellular ATP concentration in *S. albulus* M-Z18 was not caused by the acceleration of ATP synthesis rate, but because the inhibition of cell growth by lower environmental pH reduced the consumption of intracellular ATP, resulting in its accumulation in cells (Yamanaka et al., 2010). Besides, Yamanaka et al. (2010) also demonstrated that the action of Pls requires a large amount of ATP to provide energy, while the lower environmental pH can lead to the accumulation of intracellular ATP, which provides sufficient energy for the activity of Pls.

#### Effects of Environmental pH on Intracellular Free Amino Acid Concentration

**FIGURE 5:**
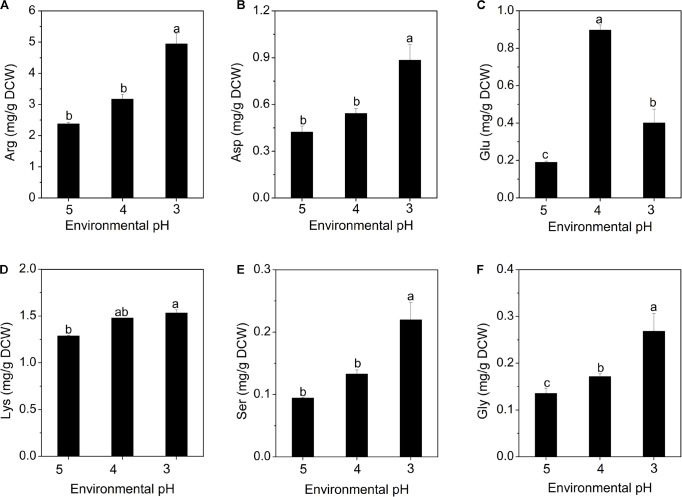
Changes in intracellular amino acid concentrations of *S. albulus* M-Z18 in batch fermentations at different environmental pH (pH 5.0, 4.0, and 3.0). Arginine **(A)**, aspartate **(B)**, glutamate **(C)**, lysine **(D)**, serine **(E),** and glycine **(F)**. Samples were collected at 27 h. Statistical significance is denoted by different letter for the same indicator.

Amino acids play important roles in the microbial resistance to acid stress, including regulation of pH_i_, generation of metabolic energy and redox power (Fernández and Zúñiga, 2006; Lund et al., 2014). As shown in Figure 5, only the concentrations of arginine, glutamate, aspartate, lysine, serine and glycine showed increasing trends with the decrease of environmental pH. Figure 5A shows that arginine accounts for the highest proportion of the intracellular free amino acids. In fact, the arginine deaminase (ADI) system is considered to be an important factor to protect microbial cells against acidic environment (Senouci-Rezkallah et al., 2011; Wu et al., 2018). Besides, aspartate can be converted into arginine to participate in the ADI system, accompanied by the formation of NH_3_ (Fernández and Zúñiga, 2006). Moreover, aspartate can also form alanine to consume the intracellular proton. Likewise, aspartate was also found to enhance the resistance of bacteria to acidic environment in studies by Wu et al. (2012a) and Guan et al. (2013).

It has been reported that amino acid decarboxylase functions to maintain pH_i_ by consuming intracellular protons as part of the decarboxylation reaction (Cotter and Hill, 2003; Lund et al., 2014). When the microorganism faces acidic environment, extracellular glutamate is transported to intracellular cytoplasm through a specific transporter, and converted into γ-aminobutyrate (GABA) and CO_2_ by glutamate decarboxylase (GAD), while consuming intracellular proton, subsequently, the synthesized GABA is released extracellularly by the antiporter. This process effectively reduces the concentration of intracellular proton and slows the acidification of the cytoplasm. Besides, GABA is less acidic than glutamate, this process also leads to the alkalization of environmental pH (Biase and Pennacchietti, 2012). The glutamate content increased when pH declined to 4.0. As the acid stress level increased to pH 3.0, it dropped sharply, because glutamate was rapidly consumed to combat the extreme acid stress (Figure 5C). The GAD system has also been found to play a vital role in resisting the acidic environment in many different bacteria (Cotter et al., 2001; Senouci-Rezkallah et al., 2011; Biase and Pennacchietti, 2012; Lund et al., 2014). Many studies have shown that lysine can also consume intracellular proton by the action of lysine decarboxylase, maintain pH_i_ stability and enhance cell resistance to acid stress (Rhee et al., 2002; Senouci-Rezkallah et al., 2011). This study first reported that the acid resistance mechanism of *S. albulus* may be related to the accumulation of intracellular serine and glycine (Figures 5E,F), which may be also the action of decarboxylase. Besides, the accumulation of intracellular aspartate, glutamate and lysine is also beneficial for the biosynthesis of ε-PL, because lysine is a precursor of ε-PL, aspartate is a precursor of lysine, while glutamate provides an amino group for the biosynthesis of lysine (Yamanaka et al., 2008; Takehara et al., 2010).

### Transcriptional Analysis

#### Screening and Cluster Analysis of the ATR Genes

To further disclose the global changes of *S. albulus* M-Z18 at transcriptional level under acid stress, a comprehensive RNA-sequencing analysis was employed. To explore the ATR genes, i.e., genes synchronously up-regulated or down-regulated with the decrease of environmental pH, we further examined the intersection of DEGs in the two comparison groups (pH 5.0 vs. pH 4.0, pH 4.0 vs. pH 3.0). The results showed that there were 350 shared DEGs, including 157 (44.86%) synchronously up-regulated genes and 121 (34.57%) synchronously down-regulated genes (Supplementary Material 2). In the ATR genes, there were 97 genes with clear functional annotations were selected, of which 33 (34.02%) were associate with transcriptional regulation, 11 (11.34%) were associated with stress-response protein, 16 (16.49%) were associated with transporter, 10 (10.31%) were associated with cell envelope, 15 (15.46%) were associated with secondary metabolite biosynthesis, 6 (6.19%) were associated with DNA and RNA metabolism, and 6 (6.19%) were associated with ribosome subunit (Supplementary Figure S1). Within the ATR genes, those assigned to transcriptional regulation, transporter and secondary metabolite biosynthesis were in majority, indicating that *S. albulus* M-Z18 mainly responded to acid stress through transcriptional regulation, substance transport and secondary metabolite biosynthesis.

#### Transcriptional Regulation

Bacteria mainly employ two kinds of signal transduction system to sense and respond to environmental stresses: two-component system (TCS) and extracytoplasmic function (ECF) σ factor. The two systems are functionally similar because they usually regulate gene expression by a membrane protein (a sensor kinase or an anti-σ factor) as a pressure sensor and a transcription factor (a response regulator or an σ factor) (Hutchings et al., 2004; Capra and Laub, 2012; Mascher, 2013).

As shown in Table 1, 17 TCS and 6σ factor genes were found to respond to acid stress, of which *mprA/B*, *pepD*, *mtrA/B, sigE, and hrdD* were identified and significantly up-regulated (except MAGL000280). The genes *mprA/B*, *mtrA/B*, and *pepD* encode the TCSs of MprAB and MtrAB and an HtrA-like serine protease PepD, respectively. The *hrdD and sigE* severally encode σ factor HrdD and an ECF σ factor SigE. Moreover, two genes (MAGL005109 and MAGL004663) were annotated as PepD, three genes (MAGL007600, MAGL004990, and MAGL000280) were annotated as MtrA, and 5 genes (MAGL005608, MAGL004993, MAGL004675, MAGL004383, and MAGL008145) were annotated as SigE.

**TABLE 1:** Identification and classification of ATR genes of *S. albulus* M-Z18.

Category	Classification	Gene ID	Log 2 ratio (pH5 vs pH4)	Log 2 ratio (pH4 vs pH3)	Gene annotation
Transcriptional regulation	Two-component system	MAGL005110	1.22	4.00	OmpR family, response regulator MprA
		MAGL005111	0.90	4.13	OmpR family, sensor histidine kinase MprB
		MAGL005109	3.74	4.82	OmpR family, putative serine protease PepD
		MAGL004663	2.89	2.03	OmpR family, putative serine protease PepD
		MAGL007601	1.15	1.35	OmpR family, sensor histidine kinase MtrB
		MAGL007600	1.21	1.95	OmpR family, response regulator MtrA
		MAGL004990	1.01	2.09	OmpR family, response regulator MtrA
		MAGL000280	–2.17	–1.03	OmpR family, response regulator MtrA
		MAGL001400	1.36	3.63	OmpR family, sensor histidine kinase
		MAGL001401	1.83	4.29	OmpR family, response regulator
		MAGL008494	1.22	3.20	OmpR family, response regulator
		MAGL008493	1.30	2.61	OmpR family, sensor histidine kinase (phosphorelay)
		MAGL004989	1.02	1.55	OmpR family, sensor histidine kinase (phosphorelay)
		MAGL007479	1.24	1.32	OmpR family, sensor histidine kinase (phosphorelay)
		MAGL007903	1.23	3.04	CitB family, citrate lyase subunit beta/citryl-CoA lyase CitE
		MAGL005334	–1.04	–3.50	Sensor-like histidine kinase
		MAGL000954	–1.24	–2.87	NarL family, response regulator
	δ factor	MAGL005608	3.08	2.89	Sigma-70 factor, ECF subfamily (SIG3.2, SigE)
		MAGL004993	1.19	3.50	Sigma-70 factor, ECF subfamily (SIG3.2, SigE)
		MAGL004675	1.18	3.49	Sigma-70 factor, ECF subfamily (SIG3.2, SigE)
		MAGL004383	2.00	2.43	Sigma-70 factor, ECF subfamily (SIG3.2, SigE)
		MAGL008145	1.06	1.45	Sigma-70 factor, ECF subfamily (SIG3.2, SigE)
		MAGL003924	0.85	3.55	RNA polymerase principal sigma factor hrdD
	Others	MAGL005988	1.37	3.69	Putative transcriptional regulator
		MAGL003720	1.30	2.93	Cell envelope-related transcriptional attenuator
		MAGL003845	3.25	2.32	AraC family transcriptional regulator
		MAGL003492	–1.54	–2.67	AraC family transcriptional regulator, transcriptional activator FtrA
		MAGL002768	–1.18	–2.46	MarR family transcriptional regulator
		MAGL000045	–1.11	–2.04	XRE family transcriptional regulator
		MAGL002769	–1.08	–2.05	ArsR family transcriptional regulator
		MAGL006445	–1.25	–1.33	GntR family transcriptional regulator
		MAGL001860	–1.50	–1.07	TetR family transcriptional regulator
		MAGL004604	–1.44	–1.03	PadR-like family transcriptional regulator
Stress-response protein		MAGL008579	1.29	3.12	LytR family regulatory protein
		MAGL005326	–1.22	–1.36	Cold shock protein (beta-ribbon, CspA family)
		MAGL004601	1.27	5.75	Heat shock protein HtpX
		MAGL000377	1.70	2.44	Gas vesicle synthesis protein
		MAGL000379	1.46	2.20	Gas vesicle synthesis-like protein
		MAGL000380	1.06	2.13	Gas vesicle synthesis protein
		MAGL002644	1.49	2.47	Tellurium resistance protein TerZ
		MAGL008582	1.14	2.52	Tellurium resistance protein TerD
		MAGL007368	1.44	3.75	Dynein regulation protein LC7
		MAGL005335	–1.59	–3.22	Dynein regulation protein LC7
		MAGL003269	–1.23	–2.54	Dynein regulation protein LC7
Transporter	ABC transporter	MAGL007613	3.68	4.03	Putative ABC transport system ATP-binding protein, ABC.CD.A
		MAGL001674	1.98	2.10	ATP-binding cassette, subfamily C, bacterial, ABCC-BAC
		MAGL001672	1.49	1.89	ATP-binding cassette, subfamily C, bacterial, ABCC-BAC
		MAGL003784	1.44	1.67	Putative ABC transport system permease protein, ABC.CD.P
		MAGL004136	1.19	1.22	ABC-2 type transport system ATP-binding protein, ABC-2.A
		MAGL006350	–4.07	–1.57	ATP-binding cassette, subfamily B, bacterial, ABCB-BAC
		MAGL006351	–3.85	–1.18	ATP-binding cassette, subfamily B, bacterial, ABCB-BAC
		MAGL006777	–1.39	–3.60	ABC transporter permease
		MAGL001711	–1.35	–1.97	molybdenum ABC transporter periplasmic molybdate-binding protein
		MAGL001223	–1.29	–1.92	ABC transporter substrate-binding protein
		MAGL001222	–1.27	–1.91	ABC transporter permease
	ATPase family	MAGL005340	1.45	1.06	Putative integral membrane ATPase, cation transport
	MFS transporter	MAGL000518	2.37	2.40	DHA2 family, methyl viologen resistance protein, SmvA
		MAGL007840	1.02	1.15	Major facilitator superfamily protein
	Others	MAGL001413	–1.49	–1.63	EmrB/QacA family drug resistance transporter
		MAGL007618	3.20	2.17	High-affinity nickel-transport protein, nixA
Cell envelope	Cell wall	MAGL003360	2.82	3.45	peptidoglycan glycosyltransferase
		MAGL005635	2.51	2.90	peptidoglycan glycosyltransferase
		MAGL005889	1.95	3.39	peptidoglycan-based cell wall biogenesis
		MAGL008231	1.22	2.97	UDP-N-acetylmuramate dehydrogenase
		MAGL008859	1.70	2.10	N-acetylmuramoyl-L-alanine amidase
		MAGL005056	1.56	1.16	UDP-N-acetylmuramate dehydrogenase
		MAGL008821	1.32	1.71	D-alanyl-D-alanine carboxypeptidase
		MAGL003432	1.18	2.99	rod shape-determining protein MreB and related proteins
	Cell membrane	MAGL007297	1.32	1.48	Cyclopropane-fatty-acyl-phospholipid synthase
		MAGL006010	1.55	3.35	Oleoyl-ACP hydrolase
Secondary metabolite biosynthesis	Non-ribosomal peptide synthetase	MAGL007259	2.60	2.56	ε-poly-L-lysine synthetase
		MAGL006295	2.84	1.28	Non-ribosomal peptide synthetase
		MAGL007555	–1.39	–2.13	ε-poly-L-lysine-degrading enzyme
	Polyketide synthase	MAGL000332	2.12	2.82	Type I polyketide synthase AVES
		MAGL006012	1.62	2.49	Putative type I polyketide synthase
		MAGL008464	1.37	1.94	Chalcone synthase
		MAGL006345	–4.39	–1.81	Type I polyketide synthase AVES
		MAGL006341	–4.68	–1.26	Type I polyketide synthase AVES
		MAGL006344	–4.46	–1.30	Type I polyketide synthase, erythronolide synthase
		MAGL006347	–4.30	–1.15	Type I polyketide synthase, erythronolide synthase
		MAGL006343	–4.18	–1.24	Type I polyketide synthase AVES
		MAGL006322	–4.07	–1.30	Polyketide synthase 12
		MAGL006315	–4.28	–1.07	Polyketide synthase 17
		MAGL006329	–3.72	–1.11	Type I polyketide synthase, erythronolide synthase
		MAGL008328	–1.36	–1.68	Type I polyketide synthase, macrolide glycosyltransferase
DNA and RNA metabolism	DNA	MAGL003442	1.08	4.16	Deoxyribose-phosphate aldolase
		MAGL001468	–1.67	–2.63	Guanine deaminase
		MAGL001558	–1.00	–2.34	Adenosine deaminase
		MAGL004280	–1.02	–1.56	Phosphoribosylformylglycinamidine synthase
		MAGL004278	–1.06	–1.43	Phosphoribosylformylglycinamidine synthase
	RNA	MAGL003493	–1.23	–1.65	Oligoribonuclease
Ribosome subunit		MAGL004745	–4.11	–2.38	Large subunit ribosomal protein L32
		MAGL004097	–1.23	–2.47	Large subunit ribosomal protein L7/L12
		MAGL004098	–1.04	–2.34	Large subunit ribosomal protein L10
		MAGL003821	–1.10	–1.80	Large subunit ribosomal protein L25
		MAGL004084	–1.01	–1.40	Large subunit ribosomal protein L2
		MAGL004558	–1.04	–1.12	Large subunit ribosomal protein L9

In *Streptomyces* species, SigE is a key regulator of the cell envelope stress response, which activated a complex regulatory network. The *sigE* gene locates in a four-gene operon, *sigE cseA cseB cseC*, with *cseA* encoding a lipoprotein CseA (negative regulator), *cseB* encoding a response regulator CseB and *cseC* encoding a membrane-anchored sensor kinase CseC. The transcription of SigE is not regulated by an anti-σ factor but completely controlled by the TCS, CseBC. Moreover, > 90% of transcription terminates directly downstream of the *sigE* gene (Tran et al., 2019). Therefore, the transcription levels of *cseB* and *cseC* show no significant difference in most instances. HrdD was reported to show the most sensitive response to pH changes, and the transcription of *hrdD* increased under acidic pH shock (Kim et al., 2008). In addition, Wang et al. (2015) proved that HrdD can specifically bind to the promoter of the *pls* (Pls gene), so it might regulate the transcription of *pls* and initiate the biosynthesis of ε-PL. Besides, SigE identifies the promoter of *hrdD* and regulates the transcription of *hrdD* (Paget et al., 1999). Therefore, it is deduced that SigE could regulate the transcription of *pls* through HrdD.

The function of MprAB and PepD was scarcely reported in *Streptomyces* species. In *Mycobacterium* species, MprAB and PepD together constitute a signal transduction system and *mprA/B* locates immediately upstream of *pepD* (White et al., 2010). MprAB positively regulates the expression of *pepD* and *sigE* to respond to membrane stress. Besides, the transcription of *pepD* is regulated by SigE and the deletion of *pepD* or *mprA/B* upregulated the expression of *sigE* (He et al., 2006; Pang et al., 2007; White et al., 2010). However, there were some differences between both species, e.g., the *pepD* (MAGL005109) was located immediately upstream of *mprA* (MAGL005110) and *mprB* (MAGL005111) in *S. albulus* M-Z18, the transcription of *sigE* is completely controlled by the CseBC and the SigE regulon does not include *pepD* in *Streptomyces* (Tran et al., 2019). Notably, Pan L. et al. (2019) proved that the signal transduction system of MprAB and PepD in *S. albulus* can regulate the transcription of *pls*.

The other TCS MtrAB is highly conserved in actinobacteria and plays pleiotropic roles in cell cycle progression, morphology, antibiotic resistance, secondary metabolite production, osmoprotection and substance transport (Som et al., 2017; Zhang et al., 2017; Pan Q. et al., 2019). Besides, 10 transcriptional regulators were also identified, which were mainly from AraC, MarR, XRE, ArsR, GntR, TetR, and PadR-like families. However, the specific functions of these genes on the ATR of *S. albulus* M-Z18 were not clear in this study.

#### Stress-Response Protein

As summarized in Table 1, 11 genes were expressed as stress-response proteins. LytR family protein is predicted to be involved in cell wall teichoic acid deposition, which is controlled by SigE (Tran et al., 2019). The gene (*lytR*, MAGL008579) of LytR family regulatory protein was up-regulated under acid stress to stabilize cell wall. The cold shock protein of CspA family can be expressed at low temperature. As a chaperone of RNA, it can prevent mRNA from forming a stable secondary structure at low temperature, ensuring the transcription and translation of genes at low temperature (Jiang et al., 1996). However, the transcription of *cspA* (MAGL005326) was down-regulated with the decline of environmental pH in this study, which could facilitate mRNA to form a stable secondary structure to prevent degrading. Therefore, when the environmental pH returned to the normal range, these mRNAs can resume function. HtpX is a protein degradation enzyme located on the cell membrane, which plays an important role in the quality control of integral membrane proteins (Sakoh et al., 2005). Kim et al. (2008) found that the expression of gas vesicle synthesis protein can be up-regulated by acidic pH shock in *S. coelicolor*. Likewise, the three genes (MAGL000377, MAGL000379, and MAGL000380) of gas vesicle synthesis protein were also found to be up-regulated under acid stress, which may be related to the ATR of *Streptomyces*.

#### Transporter

In response to acid stress, 16 ATR genes associated with transporter were detected (Table 1). Eleven genes were annotated as ATP-binding cassette (ABC) transporters, 1 membrane ATPase gene, 2 major facilitator superfamily (MFS) transporter genes and another 2 genes were annotated as EmrB/QacA family drug resistance transporter and high-affinity nickel-transport protein, respectively. The ABC transporter is composed of importer and exporter, which is responsible for the intake of nutrients and secretion of intracellular substances (mainly secondary metabolites). Besides, ABC transporters belong to the primary active transporters, they can consume ATP to transport small and large molecules (Davidson and Maloney, 2007). Among the ATR genes of transporter, the ABC transporter genes account for the most, in which 5 genes were up-regulated. Taken together, the ABC transporters of *S. albulus* M-Z18 could response to acid stress by uptake of nutrients such as amino acids and excretion of secondary metabolites, e.g., ε-PL. Notably, the gene (MAGL005340) of membrane ATPase functioned to transport cation was found to be up-regulated. It is reported that cation influx through membrane cation ATPase was an important factor in adaptation to weak-acid stress by food spoilage yeasts (Macpherson et al., 2005). Therefore, we speculated that this cation ATPase may play the same role in *S. albulus* M-Z18.

#### Cell Envelope

Acid stress significantly affected the transcription of cell envelope genes (Table 1). Seven genes (MAGL003360, MAGL005635, MAGL005889, MAGL008231, MAGL008859, MAGL005056, and MAGL008821) related to peptidoglycan synthesis were all up-regulated. The gene MAGL003432 encoding MreB was also up-regulated. In the rod-shaped bacteria like *E. coli* and *B. subtilis*, MreB acts to direct peptidoglycan biosynthesis in the lateral wall (Errington, 2015). Unlike the rod-shaped bacteria, *Streptomyces* hyphal growth at the tip does not require MreB but is directed by a polarisome complex involving DivIVA, Scy and FilP (Bush et al., 2015). However, the MreB directs to thicken the spore wall, which makes *Streptomyces* spores resistant to detrimental environmental conditions (Kleinschnitz et al., 2011). Besides, the gene *mreB* is also a target of SigE (Tran et al., 2019). Thus, SigE might regulate MreB to direct cell wall thickening through peptidoglycan biosynthesis, when *Streptomyces* suffered acid stress. Besides, the ATR genes (*cfa*, MAGL007297 and *olah*, MAGL006010) of cyclopropane-fatty-acyl-phospholipid synthase (EC 2.1.1.79) and oleoyl-ACP hydrolase (EC 3.1.2.14) were all up-regulated, which would facilitate the biosynthesis of CFA and C_18__:__1_ to enhance the ATR of the cells.

#### Secondary Metabolite Biosynthesis

In actinomycetes, the synthesis of secondary metabolites is catalyzed by a variety of enzyme systems, the most important of which are polyketide synthase (PKS) and non-ribosomal peptide synthase (NRPS). Table 1 lists that the transcription of two NRPS genes was up-regulated by acid stress, one of which was the Pls gene (MAGL007259), while majority of the PKS genes (9 out of 12) were down-regulated by acid stress. It is indicated that acid stress could inhibit the expression of PKS, so that more metabolism flowed to the synthesis of ε-PL. Besides, the transcription of ε-degrading enzyme (Pld) gene (*pld*) was down-regulated. The Pld of *S. albulus* locates on cell membrane. *In vitro*, the activity of Pld is significantly inhibited by the decline of pH from 7.0 (Kito et al., 2002). In this study, we found that the transcription of *pld* was also inhibited by the decline of environmental pH.

#### DNA, RNA Metabolism and Ribosome Subunit

With acid stress, the ATR gene (MAGL003442) for DNA synthesis was up-regulated and those for DNA degradation (MAGL001468, MAGL001558, MAGL004280, and MAGL004278) were down-regulated. Similarly, oligoribonuclease gene (MAGL003493) was down-regulated, which will cooperate with the down-regulated CspA gene (*cspA*, MAGL005326) to inhibit the degradation of RNA (Table 1). These will ensure the stability of DNA and RNA under acid stress.

Besides, 6 ATR genes encoding large subunit ribosomal protein were all down-regulated (Table 1), indicating that the biosynthesis of ribosome was inhibited. The inhibition of ribosome synthesis will reduce the synthesis of intracellular protein, which restrains bacterial growth and decreases the consumption of intracellular ATP. It is in agreement with the accumulation of intracellular ATP under acid stress (Figure 4B).

#### Validation of RNA-Sequencing Using qRT-PCR

**FIGURE 6:**
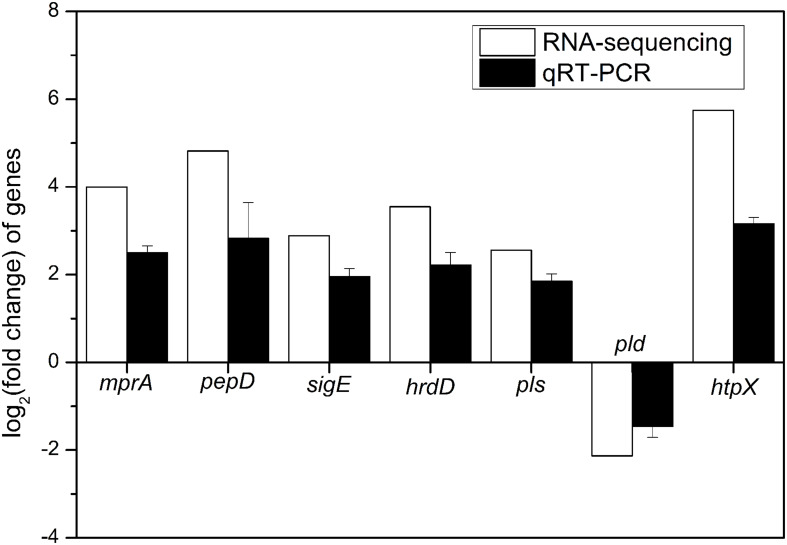
Quantitative RT-PCR validation of 7 DEGs (*mprA*, *pepD*, *sigE*, *hrdD*, *pls*, *pld*, and *htpX*) identified by RNA-sequencing in the comparison of batch fermentations by *S. albulus* M-Z18 at pH 4.0 vs. pH 3.0.

To verify the reliability of RNA-sequencing, qRT-PCR analyses of 7 DEGs (*mprA*, *pepD*, *sigE*, *hrdD*, *pls*, *pld*, and *htpX*) were performed. It is indicated that the transcription levels of 6 genes, including *mprA*, *pepD*, *sigE*, *hrdD*, *pls*, and *htpX*, were up-regulated, and that of *pld* was down-regulated (Figure 6), which were consistent with the results of RNA-sequencing.

## Conclusion

**FIGURE 7:**
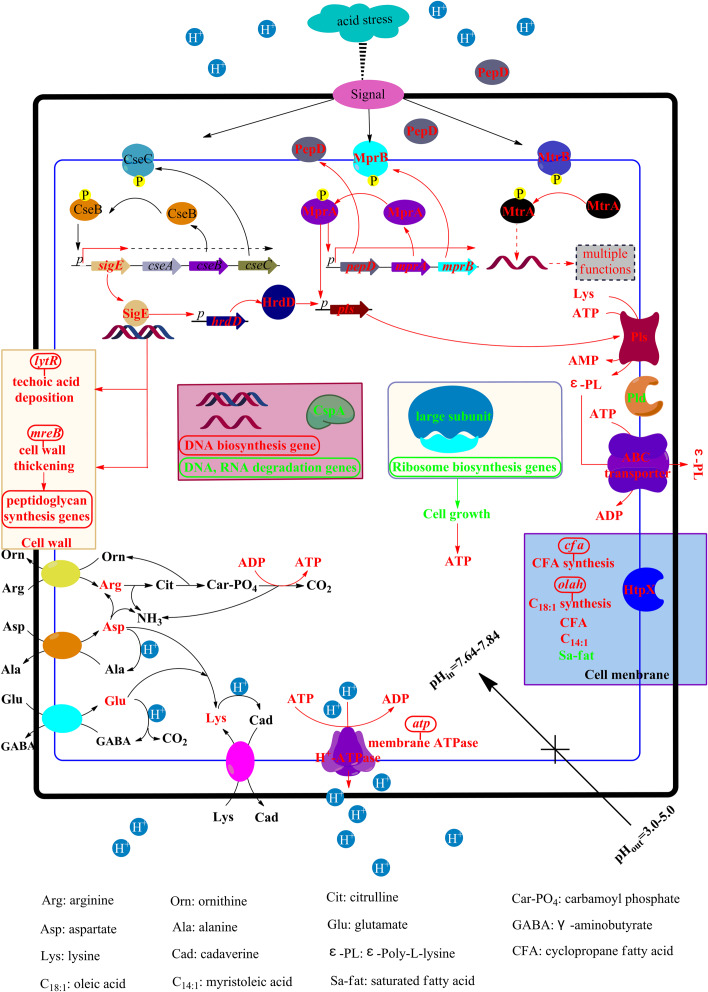
The physiological and transcriptional response mechanisms of *S. albulus* to spontaneous acid stress in the biosynthesis of ε-PL. Red represents up-regulation or increase, green represents down-regulation or decrease.

Based on the above results, the ATR of *S. albulus* was preliminarily proposed (Figure 7). To combat the spontaneous acid stress in the biosynthesis of ε-PL, *S. albulus* has developed pleietrepie response mechanisms. When *S. albulus* faced acid stress, signals originated in the cell envelope, the CseBC TCS was activated, resulting the up-regulation of *sigE*. The SigE was employed by core RNA polymerase to transcribe its regulon, including *lytR* (wall teichoic acid deposition) and *mreB* (cell wall thickening through directing peptidoglycan biosynthesis), which helped to maintain the cell wall stability. Meanwhile, the cell membrane maintained proper physiological functions through the up-regulation of related genes to increase U/S ratios, the decrease of fatty acid chain-length and the up-regulation of *htpX* to degrade or detach the mismatched proteins on cell membranes. Besides, the pH_i_ was maintained homeostasis at about 7.7: the increased intracellular amino acids, especially arginine, glutamate, aspartate and lysine, could consume more proton, generate more NH_3_ and ATP; the transcription of ribosome large subunits was down-regulated, which affects the synthesis of proteins, thus inhibiting cell growth and leading to the accumulation of intracellular ATP; the improved H^+^-ATPase activity expelled protons at the expense of consuming ATP. All of these helped to alleviate cytoplasmic acidification under acid stress. The synthesis of DNA was promoted and the degradation of DNA and RNA was suppressed, while the down-regulation of *cspA* could make it easier for RNA to form stable secondary structure to prevent degradation. In addition, the up-regulated *hrdD* under the control of SigE and the activated MprAB and PepD signal transduction system together resulted in the up-regulation of *pls*, along with the accumulated intracellular ATP, glutamate, aspartate, lysine and the suitable pH_i_, the production of ε-PL was eventually promoted. Moreover, the transcription of *pld* was also down-regulated by acid stress. Considering that ε-PL is an alkaline polymer, the synthesis of ε-PL is also deduced to be the response of *S. albulus* to acid stress.

## Data Availability Statement

RNA-seq data of *S. albulus* M-Z18 at different environmental pH values (pH 5.0, 4.0 and 3.0) were deposited at Sequence Read Archive (SRA) of National Center for Biotechnology Information (NCBI) under the accessions of SAMN14996498, SAMN14996497, and SAMN14996496, respectively.

## Author Contributions

CW and XR conceived and designed the experiments, and wrote the manuscript. CW, CY, and LW performed the experiments. CW, XR, and JW analyzed the data. XZ and XL edited and polished the manuscript. All authors contributed to the article and approved the submitted version.

## Conflict of Interest

XZ was employed by IntellectiveBio Co., Ltd. The remaining authors declare that the research was conducted in the absence of any commercial or financial relationships that could be construed as a potential conflict of interest.

## References

[B1] Biase D. D. Pennacchietti E. (2012). Glutamate decarboxylase-dependent acid resistance in orally acquired bacteria: function, distribution and biomedical implications of the *gadBC* operon. *Mol. Microbiol.* 86 770–786. 10.1111/mmi.12020 22995042

[B2] Breeuwer P. Drocourt J. L. Frank M. R. Abee T. (1996). A novel method for continuous determination of the intracellular pH in bacteria with the internally conjugated fluorescent probe 5 (and 6-)-carboxyfluorescein succinimidyl ester. *Appl. Environ. Microbiol.* 62 178–183. 10.1128/aem.62.1.178-183.1996 16535209 PMC1388751

[B3] Broadbent J. R. Larsen R. L. Deibel V. Steele J. L. (2010). Physiological and transcriptional response of *Lactobacillus casei* ATCC 334 to acid stress. *J. Bacteriol.* 192 2445–2458. 10.1128/JB.01618-09 20207759 PMC2863488

[B4] Budin-Verneuil A. Pichereau V. Auffray Y. Ehrlich D. Maguin E. (2005). Proteomic characterization of the acid tolerance response in *Lactococcus lactis* MG1363. *Proteomics* 5 4794–4807. 10.1002/pmic.200401327 16237734

[B5] Bush M. J. Tschowri N. Schlimpert S. Flärdh K. Buttner M. J. (2015). c-di-GMP signalling and the regulation of developmental transitions in streptomycetes. *Nat. Rev. Microbiol.* 13 749–760. 10.1038/nrmicro3546 26499894

[B6] Cao-Hoang L. Marechal P. A. Le-^Thanh M. Gervais P. Wache’ Y. (2008). Fluorescent probes to evaluate the physiological state and activity of microbial biocatalysts: a guide for prokaryotic and eukaryotic investigation. *Biotechnol. J.* 3 890–903. 10.1002/biot.200700206 18481263

[B7] Capra E. J. Laub M. T. (2012). Evolution of two-component signal transduction systems. *Annu. Rev. Microbiol.* 66 325–347. 10.1146/annurev-micro-092611-150039 22746333 PMC4097194

[B8] Chang Y. Y. Cronan J. E. (1999). Membrane cyclopropane fatty acid content is a major factor in acid resistance of *Escherichia coli*. *Mol. Microbiol.* 33 249–259. 10.1046/j.1365-2958.1999.01456.x 10411742

[B9] Corvini P. F. X. Gautier H. Rondags E. Vivier H. Goergen J. L. Germain P. (2000). Intracellular pH determination of pristinamycin-producing *Streptomyces pristinaespiralis* by image analysis. *Microbiology* 146 2671–2678. 10.1099/00221287-146-10-2671 11021942

[B10] Cotter P. D. Gahan C. G. Hill C. (2001). A glutamate decarboxylase system protects *Listeria monocytogenes* in gastric fluid. *Mol. Microbiol.* 40 465–475. 10.1046/j.1365-2958.2001.02398.x 11309128

[B11] Cotter P. D. Hill C. (2003). Surviving the acid test: responses of gram-positive bacteria to low pH. *Microbiol. Mol. Biol. Rev.* 67 429–453. 10.1128/mmbr.67.3.429-453.2003 12966143 PMC193868

[B12] Davidson A. L. Maloney P. C. (2007). ABC transporters: how small machines do a big job. *Trends Microbiol.* 15 448–455. 10.1016/j.tim.2007.09.005 17920277

[B13] Denich T. J. Beaudette L. A. Lee H. Trevors J. T. (2003). Effect of selected environmental and physico-chemical factors on bacterial cytoplasmic membranes. *J. Microbiol. Meth.* 52 149–182. 10.1016/S0167-7012(02)00155-0 12459238

[B14] Errington J. (2015). Bacterial morphogenesis and the enigmatic MreB helix. *Nat. Rev. Microbiol.* 13 241–248. 10.1038/nrmicro3398 25578957

[B15] Fernández M. Zúñiga M. (2006). Amino acid catabolic pathways of lactic acid bacteria. *Crit. Rev. Microbiol.* 32 155–183. 10.1080/10408410600880643 16893752

[B16] Fountoulakis M. Lahm H. W. (1998). Hydrolysis and amino acid composition analysis of proteins. *J. Chromatogr. A* 826 109–134. 10.1016/S0021-9673(98)00721-3 9917165

[B17] Fozo E. M. Quivey R. G. Jr. (2004). The *fabM* gene product of *Streptococcus mutans* is responsible for the synthesis of monounsaturated fatty acids and is necessary for survival at low pH. *J. Bacteriol.* 186 4152–4158. 10.1128/JB.186.13.4152-4158.2004 15205416 PMC421590

[B18] Guan N. Liu L. Shin H. D. Chen R. R. Zhang J. Li J. (2013). Systems-level understanding of how *Propionibacterium acidipropionici* respond to propionic acid stress at the microenvironment levels: mechanism and application. *J. Biotechnol.* 167 56–63. 10.1016/j.jbiotec.2013.06.008 23792099

[B19] Guerzoni M. E. Lanciotti R. Cocconcelli P. S. (2001). Alteration in cellular fatty acid composition as a response to salt, acid, oxidative and thermal stresses in *Lactobacillus helveticus*. *Microbiology* 147 2255–2264. 10.1099/00221287-147-8-2255 11496002

[B20] He H. Hovey R. Kane J. Singh V. Zahrt T. C. (2006). MprAB is a stress-responsive two-component system that directly regulates expression of sigma factors SigB and SigE in *Mycobacterium tuberculosis*. *J. Bacteriol.* 188 2134–2143. 10.1128/JB.188.6.2134-2143.2006 16513743 PMC1428128

[B21] Hutchings M. I. Hoskisson P. A. Chandra G. Buttner M. J. (2004). Sensing and responding to diverse extracellular signals? Analysis of the sensor kinases and response regulators of *Streptomyces coelicolor* A3(2). *Microbiology* 150 2795–2806. 10.1099/mic.0.27181-0 15347739

[B22] Hutkins R. W. Nannen N. L. (1993). pH homeostasis in lactic acid bacteria. *J. Dairy Sci.* 76 2354–2365. 10.3168/jds.S0022-0302(93)77573-6

[B23] Itzhaki R. (1972). Colorimetric method for estimating polylysine and poly-arginine. *Anal. Biochem.* 50 569–574. 10.1016/0003-2697(72)90067-X 4646067

[B24] Jiang W. Fang L. Inouye M. (1996). Complete growth inhibition of *Escherichia coli* by ribosome trapping with truncated *cspA* mRNA at low temperature. *Genes Cells* 1 965–976. 10.1046/j.1365-2443.1996.d01-219.x 9077460

[B25] Kahar P. Iwata T. Hiraki J. Park E. Y. Okabe M. (2001). Enhancement of ε-polylysine production by *Streptomyces albulus* strain 410 using pH control. *J. Biosci. Bioeng.* 91 190–194. 10.1016/S1389-1723(01)80064-5 16232973

[B26] Kim Y. J. Moon M. H. Song J. Y. Smith C. P. Hong S. K. Chang Y. K. (2008). Acidic pH shock induces the expressions of a wide range of stress-response genes. *BMC Genomics* 9:604. 10.1186/1471-2164-9-604 19087294 PMC2631018

[B27] Kito M. Takimoto R. Yoshida T. Nagasawa T. (2002). Purification and characterization of an ε-poly-L-lysine-degrading enzyme from an ε-poly-L-lysine-producing strain of *Streptomyces albulus*. *Arch. Microbiol.* 178 325–330. 10.1007/s00203-002-0459-6 12375099

[B28] Kleinschnitz E. M. Heichlinger A. Schirner K. Winkler J. Latus A. Maldener I. (2011). Proteins encoded by the mre gene cluster in *Streptomyces coelicolo*r A3(2) cooperate in spore wall synthesis. *Mol. Microbiol.* 79 1367–1379. 10.1111/j.1365-2958.2010.07529.x 21244527

[B29] Lund P. Tramonti A. Biase D. D. (2014). Coping with low pH: molecular strategies in neutralophilic bacteria. *FEMS Microbiol. Rev.* 38 1091–1125. 10.1111/1574-6976.12076 24898062

[B30] Macpherson N. Shabala L. Rooney H. Jarman M. G. Davies J. M. (2005). Plasma membrane H^+^ and K^+^ transporters are involved in the weak-acid preservative response of disparate food spoilage yeasts. *Microbiology* 151 1995–2003. 10.1099/mic.0.27502-0 15942006

[B31] Manteca A. Alvarez R. Salazar N. Yagüe P. Sanchez J. (2008). Mycelium differentiation and antibiotic production in submerged cultures of *Streptomyces coelicolor*. *Appl. Environ. Microbiol.* 74 3877–3886. 10.1128/AEM.02715-07 18441105 PMC2446541

[B32] Mascher T. (2013). Signaling diversity and evolution of extracytoplasmic function (ECF) sigma factors. *Curr. Opin. Microbiol.* 16 148–155. 10.1016/j.mib.2013.02.001 23466210

[B33] O’Sullivan E. Condon S. (1997). Intracellular pH is a major factor in the induction of tolerance to acid and other stresses in *Lactococcus lactis*. *Appl. Environ. Microb.* 63 4210–4215. 10.1128/aem.63.11.4210-4215.1997 PMC168739 9361406

[B34] Paget M. S. Chamberlin L. Atrih A. Foster S. J. Buttner M. J. (1999). Evidence that the extracytoplasmic function sigma factor σ^*E*^ is required for normal cell wall structure in *Streptomyces coelicolor* A3(2). *J. Bacteriol.* 181 204–211. 10.1128/jb.181.1.204-211.1999 9864331 PMC103550

[B35] Pan L. Chen X. Wang K. Mao Z. (2019). Understanding high ε-poly-L-lysine production by *Streptomyces albulus* using pH shock strategy in the level of transcriptomics. *J. Ind. Microbiol. Biotechnol.* 46 1781–1792. 10.1007/s10295-019-02240-z 31595454

[B36] Pan Q. Tong Y. Han Y. J. Ye B. C. (2019). Two amino acids missing of MtrA resulted in increased erythromycin level and altered phenotypes in *Saccharopolyspora erythraea*. *Appl. Microbiol. Biotechnol.* 103 4539–4548. 10.1007/s00253-019-09825-9 30997553

[B37] Pang X. Vu P. Byrd T. F. Ghanny S. Soteropoulos P. Mukamolova G. V. (2007). Evidence for complex interactions of stress-associated regulons in an mprAB deletion mutant of *Mycobacterium tuberculosis*. *Microbiology* 153(Pt 4) 1229–1242. 10.1099/mic.0.29281-0 17379732

[B38] Ren X. D. Chen X. S. Zeng X. Wang L. Tang L. Mao Z. G. (2015). Acidic pH shock induced overproduction of ε-poly-L-lysine in fed-batch fermentation by *Streptomyces* sp. M-Z18 from agro-industrial by-products. *Bioprocess Biosyst. Eng.* 38 1113–1125. 10.1007/s00449-015-1354-2 25605030

[B39] Rhee J. E. Rhee J. H. Ryu P. Y. Sang H. C. (2002). Identification of the *cadBA* operon from *Vibrio vulnificus* and its influence on survival to acid stress. *FEMS Microbiol. Lett.* 208 245–251. 10.1111/j.1574-6968.2002.tb11089.x 11959444

[B40] Rioseras B. López-García M. T. Yagüe P. Sánchez J. Manteca Á (2014). Mycelium differentiation and development of *Streptomyces coelicolor* in lab-scale bioreactors: programmed cell death, differentiation, and lysis are closely linked to undecylprodigiosin and actinorhodin production. *Bioresour. Technol.* 151 191–198. 10.1016/j.biortech.2013.10.068 24240146 PMC3858829

[B41] Russell N. J. (1984). Mechanisms of thermal adaptation in bacteria: blueprints for survival. *Trends Biochem. Sci.* 9 108–112. 10.1016/0968-0004(84)90106-3

[B42] Sakoh M. Ito K. Akiyama Y. (2005). Proteolytic activity of HtpX, a membrane-bound and stress-controlled protease from *Escherichia coli*. *J. Biol. Chem.* 280 33305–33310. 10.1074/jbc.M506180200 16076848

[B43] Sasser M. (1990). *Identification of Bacteria by Gas Chromatography of Cellular Fatty Acids. MIDI Technical Note 101.* Newark, DE: MIDI, Inc, 1–7.

[B44] Senouci-Rezkallah K. Schmitt P. Jobin M. P. (2011). Amino acids improve acid tolerance and internal pH maintenance in *Bacillus cereus* ATCC14579 strain. *Food microbiol.* 28 364–372. 10.1016/j.fm.2010.09.003 21356439

[B45] Shih I. L. Shen M. H. (2006). Optimization of cell growth and poly(ε-lysine) production in batch and fed-batch cultures by *Streptomyces albulus* IFO 14147. *Process Biochem.* 41 1644–1649. 10.1016/j.procbio.2006.03.013

[B46] Shima S. Sakai H. (1977). Polylysine produced by *Streptomyces*. *Agric. Biol. Chem.* 41 1807–1809. 10.1271/bbb1961.41.1807

[B47] Shimada S. Andou M. Naito N. Yamada N. Osumi M. Hayashi R. (1993). Effects of hydrostatic pressure on the ultrastructure and leakage of internal substances in the yeast *Saccharomyces cerevisiae*. *Appl. Microbiol. Biotechnol.* 40 123–131. 10.1007/BF00170440

[B48] Singh K. P. Mahendra A. L. Jayaraj V. Wangikar P. P. Jadhav S. (2013). Distribution of live and dead cells in pellets of an actinomycete *Amycolatopsis balhimycina* and its correlation with balhimycin productivity. *J. Ind. Microbiol. Biotechnol.* 40 235–244. 10.1007/s10295-012-1215-9 23184174

[B49] Som N. F. Heine D. Holmes N. A. Munnoch J. T. Chandra G. Seipke R. F. (2017). The conserved actinobacterial two-component system MtrAB coordinates chloramphenicol production with sporulation in *Streptomyces venezuelae* NRRL B-65442. *Front. Microbiol.* 8:1145. 10.3389/fmicb.2017.01145 28702006 PMC5487470

[B50] Takehara M. Hibino A. Saimura M. Hirohara H. (2010). High-yield production of short chain length poly(ε-L-lysine) consisting of 5-20 residues by *Streptomyces aureofaciens*, and its antimicrobial activity. *Biotechnol. Lett.* 32 1299–1303. 10.1007/s10529-010-0294-9 20464451

[B51] Ter Beek A. Wijman J. G. E. Zakrzewska A. Orij R. Smits G. J. Brul S. (2015). Comparative physiological and transcriptional analysis of weak organic acid stress in *Bacillus subtilis*. *Food Microbiol.* 45 71–82. 10.1016/j.fm.2014.02.013 25481064

[B52] Tran N. T. Huang X. Hong H. J. Bush M. J. Chandra G. Pinto D. (2019). Defining the regulon of genes controlled by σ^*E*^, a key regulator of the cell envelope stress response in *Streptomyces coelicolor*. *Mol. Microbiol.* 112 461–481. 10.1111/mmi.14250 30907454 PMC6767563

[B53] Wang L. Gao C. Tang N. Hu S. Wu Q. (2015). Identification of genetic variations associated with epsilon-poly-lysine biosynthesis in *Streptomyces albulus* ZPM by genome sequencing. *Sci. Rep.* 5:9201. 10.1038/srep09201 25776564 PMC4361855

[B54] White M. J. He H. Penoske R. M. Twining S. S. Zahrt T. C. (2010). PepD participates in the mycobacterial stress response mediated through MprAB and SigE. *J. Bacteriol.* 192 1498–1510. 10.1128/JB.01167-09 20061478 PMC2832534

[B55] Wu C. Zhang J. Chen W. Wang M. Du G. Chen J. (2012a). A combined physiological and proteomic approach to reveal lactic-acid-induced alterations in *Lactobacillus casei* Zhang and its mutant with enhanced lactic acid tolerance. *Appl. Microbiol. Biotechnol.* 93 707–722. 10.1007/s00253-011-3757-6 22159611

[B56] Wu C. Zhang J. Wang M. Du G. Chen J. (2012b). *Lactobacillus casei* combats acid stress by maintaining cell membrane functionality. *J. Ind. Microbiol. Biotechnol.* 39 1031–1039. 10.1007/s10295-012-1104-2 22366811

[B57] Wu H. Zhao Y. Du Y. Miao S. Liu J. Li Y. (2018). Quantitative proteomics of *Lactococcus lactis* F44 under cross-stress of low pH and lactate. *J. Dairy Sci.* 101 6872–6884. 10.3168/jds.2018-14594 29778478

[B58] Xu Y. Zhao Z. Tong W. Ding Y. Liu B. Shi Y. (2020). An acid-tolerance response system protecting exponentially growing *Escherichia coli*. *Nat. Commun.* 11:1496. 10.1038/s41467-020-15350-5 32198415 PMC7083825

[B59] Yamanaka K. Kito N. Imokawa Y. Maruyama C. Utagawa T. Hamano Y. (2010). Mechanism of ε-poly-L-lysine production and accumulation revealed by identification and analysis of an ε-poly-L-lysine-degrading enzyme. *Appl. Environ. Microbiol.* 76 5669–5675. 10.1128/AEM.00853-10 20601519 PMC2935060

[B60] Yamanaka K. Maruyama C. Takagi H. Hamano Y. (2008). ε-Poly-L-lysine dispersity is controlled by a highly unusual nonribosomal peptide synthetase. *Nat. Chem. Biol.* 4 766–772. 10.1038/nchembio.125 18997795

[B61] Yin Z. Feng S. Tong Y. Yang H. (2019). Adaptive mechanism of *Acidithiobacillus thiooxidans* CCTCC M 2012104 under stress during bioleaching of low-grade chalcopyrite based on physiological and comparative transcriptomic analysis. *J. Ind. Microbiol. Biotechnol.* 46 1643–1656. 10.1007/s10295-019-02224-z 31420797

[B62] Zhai Z. Yang Y. Wang H. Wang G. Ren F. Li Z. (2020). Global transcriptomic analysis of *Lactobacillus plantarum* CAUH2 in response to hydrogen peroxide stress. *Food Microbiol.* 87:103389. 10.1016/j.fm.2019.103389 31948630

[B63] Zhang P. Wu L. Zhu Y. Liu M. Wang Y. Cao G. (2017). Deletion of MtrA inhibits cellular development of *Streptomyces coelicolor* and alters expression of developmental regulatory genes. *Front. Microbiol.* 8:2013. 10.3389/fmicb.2017.02013 29085353 PMC5650626

[B64] Zhang Y. M. Rock C. O. (2008). Membrane lipid homeostasis in bacteria. *Nat. Rev. Microbiol.* 6 222–233. 10.1038/nrmicro1839 18264115

