# Supplementary material for: Physiological and Transcriptional Responses of Streptomyces albulus to Acid Stress in the Biosynthesis of ε-Poly-L-lysine
Source: Front Microbiol. 2020 Jun 19;11:1379. doi: 10.3389/fmicb.2020.01379 (PMC7317143; doi:10.3389/fmicb.2020.01379)
Supplement: Supplementary file 1 [file Data_Sheet_1.zip › Supplementary Materials/Caption for Supplementary Material 2.docx]

**Supplementary Table 2.** The intersection of differentially expressed genes in the two comparison groups of pH 5.0 vs. pH 4.0 and pH 4.0 vs. pH 3.0.
