# Supplementary material for: Physiological and Transcriptional Responses of Streptomyces albulus to Acid Stress in the Biosynthesis of ε-Poly-L-lysine
Source: Front Microbiol. 2020 Jun 19;11:1379. doi: 10.3389/fmicb.2020.01379 (PMC7317143; doi:10.3389/fmicb.2020.01379)
Supplement: Supplementary file 1 [file Data_Sheet_1.zip › Supplementary Materials/Supplementary_Material 1.docx]

Supplementary Material 1

**Supplementary Table 1.** Sequences of primer used in qRT-PCR assay.

| Gene | Gene product | Primer sequence (5’-3’) |
| --- | --- | --- |
| *mtrA* | two-component system, OmpR family, response regulator MtrA | F: GAAGCTGCTGCTCTTCCTGT  R: GCGGGGTGTCCTCTATCTTC |
| *mtrB* | two-component system, OmpR family, sensor histidine kinase MtrB | F: CTGATCGAGATCCGTGACAG  R: TGTAGAACCGGTCGAAGACG |
| *sigE* | SigE | F: ATGCTGGTGCTGCGCTACTA  R: TGCCGACACTGATGTTGAGG |
| *hrdD* | HrdD | F: TCTTCATCCGGTCCAACCT  R: GTCGCGTAGGTCGAGAACTT |
| *pls* | ε-PL synthase | F: CCCTTCTCGAATCGTCCTTC  R: GCGTTCGATCTCGATACACA |
| *pld* | ε-PL degrading enzyme | F: ACTACACCTACGCCCAGCAC  R: TCGAAGAAGACCTCGTCACC |
| *htpX* | heat shock protein | F: GTGTACGCCATCAGCTTCCT  R: TGGCTGAAGCTCTCCTTGTT |
| 16s rDNA | Reference gene | F: ACTCCTACGGGAGGCAGCAG  R: ATTACCGCGGCTGCTGG |





**Supplementary Figure 1.** Cluster analysis of the ATR genes.
